# Lost but Not Least—Novel Insights into Progesterone Receptor Loss in Estrogen Receptor-Positive Breast Cancer

**DOI:** 10.3390/cancers13194755

**Published:** 2021-09-23

**Authors:** Michał Kunc, Marta Popęda, Wojciech Biernat, Elżbieta Senkus

**Affiliations:** 1 Department of Pathomorphology, Medical University of Gdańsk, 80-214 Gdańsk, Poland; mkunc@gumed.edu.pl (M.K.); biernat@gumed.edu.pl (W.B.); 2 Laboratory of Translational Oncology, Intercollegiate Faculty of Biotechnology, Medical University of Gdańsk, 80-211 Gdańsk, Poland; marta.popeda@gumed.edu.pl; 3 Department of Oncology and Radiotherapy, Medical University of Gdańsk, 80-214 Gdańsk, Poland

**Keywords:** estrogen receptor, progesterone receptor, breast cancer, treatment, microRNA

## Abstract

**Simple Summary:**

Most breast cancers co-express estrogen receptor α (ERα) and progesterone receptor (PgR). These cancers are sensitive to endocrine therapy and, in general, have superior outcomes. However, a subset of tumors expresses ERα but loses expression of PgR in various mechanisms. The processes driving the loss of PgR may cause resistance to hormonal treatment and a more aggressive clinical course. The current review summarizes current knowledge on the biology of ERα-positive PgR(−)negative breast cancer and discusses the associations between molecular mechanisms and clinical characteristics.

**Abstract:**

Estrogen receptor α (ERα) and progesterone receptor (PgR) are crucial prognostic and predictive biomarkers that are usually co-expressed in breast cancer (BC). However, 12–24% of BCs present ERα(+)/PgR(−) phenotype at immunohistochemical evaluation. In fact, BC may either show primary PgR(−) status (in chemonaïve tumor sample), lose PgR expression during neoadjuvant treatment, or acquire PgR(−) phenotype in local relapse or metastasis. The loss of PgR expression in ERα(+) breast cancer may signify resistance to endocrine therapy and poorer outcomes. On the other hand, ERα(+)/PgR(−) BCs may have a better response to neoadjuvant chemotherapy than double-positive tumors. Loss of PgR expression may be a result of pre-transcriptional alterations (copy number loss, mutation, epigenetic modifications), decreased transcription of the *PGR* gene (e.g., by microRNAs), and post-translational modifications (e.g., phosphorylation, sumoylation). Various processes involved in the down-regulation of PgR have distinct consequences on the biology of cancer cells. Occasionally, negative PgR status detected by immunohistochemical analysis is paradoxically associated with enhanced transcriptional activity of PgR that might be inhibited by antiprogestin treatment. Identification of the mechanism of PgR loss in each patient seems challenging, yet it may provide important information on the biology of the tumor and predict its responsiveness to the therapy.

## 1. Introduction

Estrogen receptor α (ERα) and progesterone receptor (PgR) are crucial prognostic and predictive biomarkers in breast cancer (BC). Expression of steroid hormone receptors (HRs) in cancer cells justifies the introduction of endocrine therapies (ET), e.g., selective estrogen receptor modulators (SERMs), aromatase inhibitors (AIs), or selective estrogen receptor degraders (SERDs) [1]. These therapies primarily target ER, but BCs co-expressing PgR tend to show an even better response to hormonal treatment. Since the progesterone receptor gene (*PGR*) is dependent on ERα, the negative PgR status may indicate altered ERα signaling and impaired response to ET [2]. In the last two decades, the prognostic and predictive value of PgR expression has been widely disputed, with some authors postulating even to abandon PgR evaluation [3,4]. However, expression of *PGR* is included in both the 21-gene recurrence score (Oncotype DX, Genomic Health Inc., Redwood City, CA, USA) and the 50-gene signature classifying BC into the molecular intrinsic subtypes (PAM-50) [5]. Additionally, multiple studies confirmed the usefulness of joint immunohistochemical (IHC) evaluation of ERα, PgR, human epidermal growth factor receptor 2 (HER2), and Ki67, which enables subclassification of BC into surrogate intrinsic phenotypes, with the cut-off value discriminating between luminal A-like and luminal B-like tumors proposed at 20% of cells positive for PgR expression [6]. Nevertheless, according to the American Society of Clinical Oncology/College of American Pathologists (ASCO/CAP) guidelines, in routine assessment BC is considered PgR(−) if <1% or 0% of tumor cell nuclei are immunoreactive [7].

Single hormone receptor-positive breast cancers have two distinct categories. First, ERα(−)/PgR(+) BC is extraordinarily rare and is molecularly, morphologically, and clinically similar to triple-negative breast cancer [8,9]. Another type, ERα(+)/PgR(−), is relatively more common, constituting approximately 12–24% of all BC cases [10,11]. The prognostic and predictive value of this phenotype has been thoroughly analyzed and several reviews and meta-analyses have been recently published [10,12]. In general, ERα(+)/PgR(−) BCs are more often aggressive, high-grade tumors, with high proliferation index, high glucose metabolism, and outcomes inferior to double-positive tumors [13,14]. Nonetheless, patients with single hormone receptor-positive BC still benefit from hormonal therapy, and recent findings emphasize the importance of ET implementation in this group of patients [15].

ERα(+)/PgR(−) tumors develop more commonly in patients older than 55 years than the double-positive cases [10]. Lower estrogen levels in elderly females may contribute to lower expression of ERα-dependent proteins, e.g., PgR [16]. Moreover, the phase of the menstrual cycle at which the tumor is excised can influence the PgR status: carcinomas removed in the luteal phase more often display PgR(−) phenotype, compared to the follicular phase [17]. Other risk factors for ERα(+)/PgR(−) BC development include hormone replacement therapy (combination of estrogen and synthetic progestin), alcohol consumption, and some antidepressants [18,19,20].

PgR expression provides independent prognostic information and increases the prognostic accuracy of ER assessment in primary BC [21]. One study reported that the presence of PgR(+) proliferating (Ki67-expressing) cells but not PgR(+) non-proliferating cells is associated with better disease-free survival [22].

However, no effect of PgR expression on the benefit from tamoxifen use was demonstrated in the meta-analysis of 20 trials involving more than 21 thousand early BC patients [23]. In metastatic ER(+) disease, PgR expression is associated with an increased probability of response to tamoxifen, longer time to treatment failure, and longer overall survival [24]. No difference was seen, however, in the magnitude of benefits from the addition of cyclin-dependent kinases 4 and 6 (CDK4/6) inhibitor to ET for advanced BC treatment [25].

On the other hand, PgR-negativity in ERα(+) BC is associated with higher rates of pathological complete response to neoadjuvant chemotherapy (NAC) when compared to double-positive BC [26,27,28]. Thus, PgR status may be of great importance in predicting response to NAC in ERα(+) patients.

Moreover, PgR is a predictive factor (as depletion of PgR correlates with poor response to megestrol acetate in advanced BC) and a potential target for personalized therapy in BC, either with the use of antiprogestins or, surprisingly, progestogens [29].

While the epidemiology and clinical behavior of this type of single hormone receptor-positive BC is well described, the underlying biology of these tumors remains obscure. In 2005 a comprehensive description of the biology of PgR loss in ERα(+) BC was published by Cui et al. [30]. The current paper aims to provide an update on this subject, focusing on the studies published in the last 15 years. A special emphasis is put on the novel mechanisms of PgR loss, genetic landscape and biology of ERα(+)/PgR(−) tumors, and the role of microRNA (miRNA) in the down-regulation of PgR.

## 2. Mechanisms of PgR Negativity

BC may either show primary PgR negative phenotype (i.e., negative PgR expression in tumor sample assessed before systemic therapy), lose PgR expression during neoadjuvant treatment (assessed in the postsurgical specimen), or acquire PgR negative phenotype in local relapse or metastasis.

### 2.1. Loss of PgR at the Genetic Level

Among the HER2(−) group of tumors, the ERα(+)/PgR(−) cases show significantly lower *PGR* mRNA expression when compared to ER(+)/PgR(+) cancers, suggesting that in most cases the loss of PgR occurs before or during transcription [31]. At the genetic level, PgR loss might be explained by a copy number loss of the *PGR* gene, which was reported to occur in 27–52% of cases of BC [31]. Importantly, exogenous expression of PgR in breast cancer cells ensued growth inhibition in an MCF-7 cell line with a heterozygous loss of the *PGR* gene [32].

On the other hand, *PGR* mutations are exceedingly rare, since in the analysis of 959 ER(+)/PgR(−) cases all the tumors were classified as *PGR*-wild-type [33]. In another large dataset, only 9 missense mutations in the *PGR* gene were identified (estimated frequency 0.36%) [34]. A recent study on *PGR* variants in metastatic ER(+) BC demonstrated that 3 out of 4 samples of functionally deleterious Y890C variant were PgR(−) by IHC, so this specific variant may contribute to PgR loss by clonal selection [35].

### 2.2. The Interplay between Growth Factors and PgR Expression

**Figure 1:**
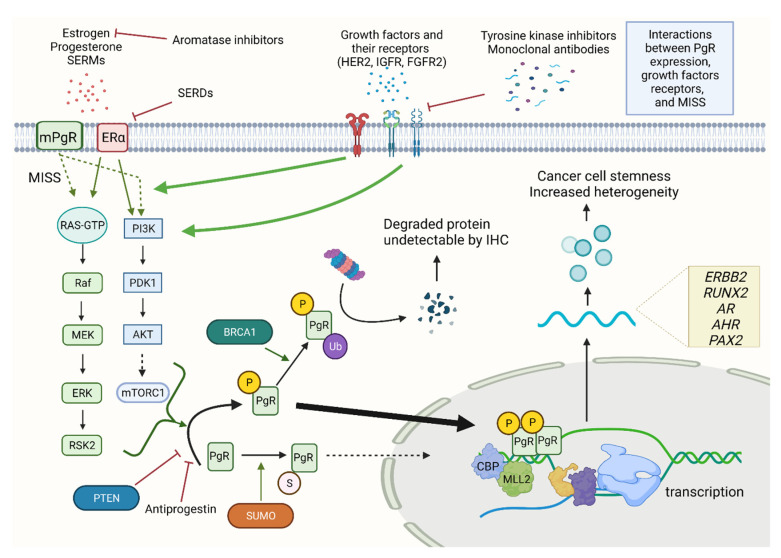
Interactions between PgR, growth factor-dependent signaling and MISS Green arrows demonstrate stimulatory effects, red T-shaped lines depict inhibition. Overactive growth factors receptors stimulate MISS and directly activate various signaling pathways leading to activation of multiple kinases, i.e., ERK, AKT, RSK2, mTORC1, which phosphorylate PgR at Ser294. Phosphorylated PgR is undersumoylated, undergoes rapid ubiquitination and degradation in proteasomes reflected by PgR(−) status in immunohistochemistry. Phosphorylated PgR is also transcriptionally overactive, recruits CBP and MLL2, and enhances transcription of genes involved in cancer progression. Abbreviations: *AHR*—aryl hydrocarbon receptor; AKT—protein kinase B; *AR*—androgen receptor; BRCA1—Breast cancer type 1 susceptibility protein; CBP—CREB-binding protein; ERα—estrogen receptor α; *ERBB2*—Erb-B2 Receptor Tyrosine Kinase 2; ERK—extracellular-regulated kinase; FGFR2—fibroblast growth factor receptor 2; HER2—human epidermal growth factor receptor 2; IGFR—insulin-like growth factor receptor; IHC—immunohistochemistry; MEK—mitogen-activated protein kinase; MISS—membrane-initiated steroid signaling; MLL2—mixed linage leukemia gene 2; mTORC1—mammalian target of rapamycin complex 1; P—phosphate residues; (m)PgR—(membranous) progesterone receptor; *PAX2*—paired box 2; Raf—rapidly accelerated fibrosarcoma; PDK1—3-phosphoinositide-dependent protein kinase-1; PI3K—phosphoinositide 3-kinase; PTEN—phosphatase and tensin homolog; RAS—rat sarcoma virus; RSK2—ribosomal S6 kinase 2; *RUNX2*—RUNX Family Transcription Factor 2; SERDs—selective estrogen receptor degraders; SERMs—selective estrogen receptor modulators; Ub—ubiquitin. Created with BioRender.com—accessed date 22 September 2021.

The role of growth factors and growth factors receptors in the pathogenesis of ERα(+)/PgR(−) tumors has been postulated for many years [30]. Insulin-like growth factor (IGF), epidermal growth factor (EGF), and heregulin activate signaling pathways down-regulating PgR expression [30]. Accordingly, ERα(+)/PgR(−) BCs demonstrate an increased frequency of epidermal growth factor receptor (EGFR) and HER2 overexpression [30]. In normal circumstances, ERα mainly exerts genomic effects but in the case of enhanced growth factor stimulation, membrane-initiated steroid signaling (MISS) starts to predominate [26]. This transition ensues PgR down-regulation by its phosphorylation via extracellular signal-regulated protein kinase (ERK1/2), phosphatidylinositol 3-kinase (PI3K), Akt, and mammalian target of rapamycin complex 1 (mTORC1) (Figure 1). Importantly, SERMs can stimulate MISS, which partially explains the greater benefits of ERα(+)/PgR(−) patients from AIs treatment compared to tamoxifen [30,36].

Additional proofs of the role of growth factors in the development of ERα(+)/PgR(−) BC come from a neu-related lipocalin-transforming growth factor α (NRL-TGFα) transgenic mouse model [37]. During tumorigenesis, ERα expression was noted in all types of precursor lesions and persisted in cancer, whereas PgR expression was lost very early. In bi-transgenic mice overexpressing prolactin (PRL) and TGFα (NRL-PRL/TGFα), these hormones cooperatively enhance Akt activity, resulting in decreased PgR and increased ERα expression [38]. Despite enhanced ERα expression, the developed tumors were insensitive to estrogens, again supporting the hypothesis on diminished hormone responsiveness in ERα(+)/PgR(−) BC. Thus, targeting growth factors pathways may increase sensitivity to ET in single hormone receptor-positive BC.

### 2.3. Molecular Mechanisms Underlying False-Negative PgR Staining in IHC

Progesterone receptor undergoes multiple post-translational modifications, including phosphorylation, acetylation, sumoylation, methylation, and ubiquitination [39]. Even in the absence of ligands, PgR is constitutively phosphorylated at some sites, and exposure to progestogen results in a net increase in the phosphorylation [40]. The result of this modification depends on a specific phosphorylation site that modulates PgR stability, nuclear transport, DNA binding, and transcriptional activity. Hormone binding results in poly-ubiquitination of PgR leading to ligand-induced PgR down-regulation—this process is paradoxically the hallmark of cells actively expressing PgR-dependent genes [40]. In human BC cells, ERK1/2 activation triggers PgR-B phosphorylation at Ser294, which, thereby, inhibits PgR sumoylation at Lys388. Undersumoylated PgR(−)B is derepressed and transcriptionally overactive, thus highly sensitive to low progestin concentration [41] (Figure 1). However, Ser294 phosphorylation targets the receptors for rapid proteasomal degradation [42]. Moreover, PgR Ser294 and Ser400 phosphorylation reduce PgR nuclear export, probably enhancing the genomic action of progesterone [43], and phosphorylation-induced PgR desumoylation enhances the transcription of proliferative genes via recruitment of a CREB-binding protein (CBP) and mixed linage leukemia gene 2 (MLL2) [44]. Thus, in the final effect, PgR might express enhanced transcriptional activity but, simultaneously, undergo instant degradation and be undetectable by IHC [42]. An animal study by Zhang et al. demonstrated that the loss of tumor suppressor, Tat-Interacting Protein (Tip30), accelerates cancerogenesis in the MMTV-Neu mouse model of BC, and leads to the development of exclusively ER(+)/PgR(−) tumors [45]. Loss of Tip30 results in impaired degradation of EGFR and enhanced Akt signaling, which correlated with both increased expression and phosphorylation of ERα and loss of PgR in IHC staining [45]. In in vitro culture, the PgR protein was detectable following proteasome inhibition, and the progesterone antagonist RU486 suppressed the growth of Neu+/Tip30−/− tumors [45].

Finally, various clones of anti-ER and anti-PgR antibodies may show discordant results, and multiple additional pre-analytic or analytic factors influence the final quantification of steroid hormones expression. Failure to detect PgR expression by IHC occurs in various laboratories with a frequency of 5 to 15% of cases [46]. While PgR-negativity assessed by IHC may be a technical issue, the other possibility is that alternative splicing of PgR produces cancer-specific variants of PgR that are undetectable with N-terminally targeting antibodies. These truncated variants are generated by the deletion of some of the eight exons of *PGR* or by the preservation of introns and are capable of binding to progesterone, interacting with co-factors, and binding to DNA, thus they may remain functional [47]. Nevertheless, the clinical significance of alternative splicing of PgR needs to be elucidated. Identification of patients with false-negative PgR status may help to identify patients who are more likely to benefit from ET.

### 2.4. Influence of Tumor Suppressors Loss on PgR Expression

The phosphatase and tensin homolog (PTEN) is a tumor suppressor frequently lost in BC [48]. The role of PTEN is to dephosphorylate phosphatidylinositol 3,4,5-triphosphate (PIP3), thus the loss of PTEN correlates with higher levels of PIP3, which, in turn, activates the Akt signaling pathway [48]. Loss of heterozygosity at the *PTEN* locus coexisting with HER2 overexpression results in substantial Akt activation, leading to loss of PgR [49] (Figure 1). Additionally, PTEN-knockout mice (K8PTEN-KO) demonstrate increased proliferation of mammary epithelial cells mainly restricted to the preferential expansion of PgR(−) cells [50].

In contrast to PTEN, the association between Breast cancer type 1 susceptibility protein (BRCA1) and PgR expression is ambiguous. On the one hand, BRCA1 was reported to stimulate the ubiquitination of PgR protein by E2 enzyme UbcH5c and its subsequent degradation [51]. On the other hand, Sanford et al. found no difference in the proportion of low-positive (<10% positive cells) and negative PgR staining between patients with and without deleterious germline *BRCA1* mutations [52].

### 2.5. Epigenetic Mechanisms of PgR Suppression

DNA methylation is the most important epigenetic mechanism orchestrating transcription. The first report on the inverse association between *PGR* promoter methylation and PgR expression in BC was published in 1996 and since then this observation has been confirmed by several studies [53]. Recent data demonstrate that IHC PgR(−) tumors show higher *PGR* methylation [54,55,56,57]. Nonetheless, in PgR(−) breast tumors, *PGR* methylation is usually either low or absent, so hypermethylation of *PGR* promoter is unlikely the major mechanism of PgR silencing, albeit some data are contradictory [56,57,58]. Interestingly, one study reported a higher incidence of DNA methylation in *PGR* promoter in HER2-amplified/overexpressing cases, pointing to the role of methylation in the pathogenesis of ER(+)/PgR(−)/HER2(+) breast tumors [59].

Several studies point to an association between *PGR* methylation and patients’ outcome, e.g., tamoxifen resistance [57,60]. Additionally, long-term tamoxifen treatment leads to epigenetic silencing of ER-responsive genes, including *PGR* [61]. Owing to a high prevalence of ER(+)PgR(−) phenotype among breast tumors recurring after tamoxifen treatment, *PGR* methylation status was proposed as a predictive marker for tamoxifen insensitivity [61]. Consequently, loss of PgR was also demonstrated in BC cell lines with decreased tamoxifen sensitivity following long-term treatment [62]. Moreover, in MCF-7 BC cell line signaling from membrane-associated ER contributes to epigenetic modulation of *PGR* gene via the action of histone methyltransferase enhancer of Zeste homolog 2-EZH2 [63].

Numerous groups have reported on the restoration of *PGR* gene expression in PgR(−) cell lines following treatment with agents blocking DNA epigenetic modifications, namely the inhibitors of histone deacetylases and DNA methyltransferases [64,65]. Exposure to epigenetic modulators also resulted in increased *PGR* mRNA expression in the hormone-receptor-positive MCF-7 cell line [64]. In the future, it may be possible to convert PgR(−) BC into PgR(+) with the use of epigenetic modulators in order to enhance its sensitivity to ET [66].

### 2.6. The Interplay between Isoforms and Splice Variants of Steroid Hormone Receptors and PgR Expression

**Figure 2:**
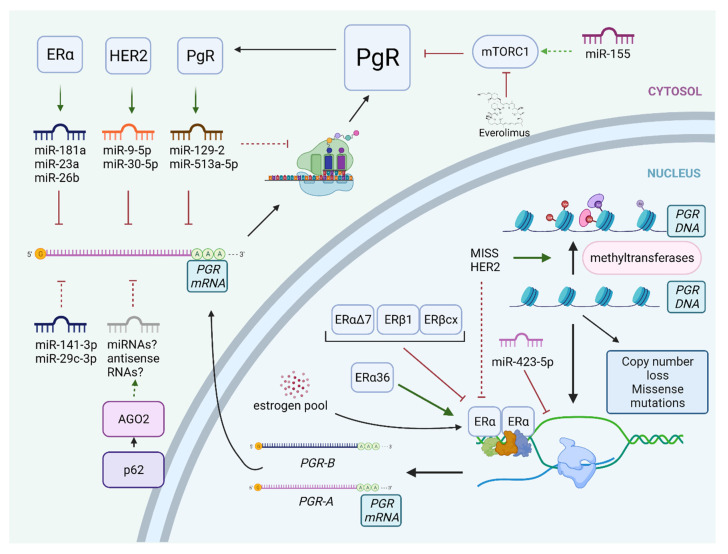
Pre-translational mechanisms of PgR loss and down-regulation. Green arrows indicate stimulatory effects, red T-shaped lines depict inhibitory effects, dotted lines show potential effects. At pre-transcriptional stage, PgR loss is a consequence of methylation of *PGR* promoter, copy number loss (often), or mutations (very rarely). Splice variants of ERα and ERβ may either suppress or activate the transcription of *PGR*. Low levels of estradiol after menopause are frequently insufficient to induce expression of PgR. *PGR* mRNA is a direct target of multiple miRNAs, but some miRNAs may down-regulate PgR indirectly, e.g., via activation of mTORC1. For details, see text. Abbreviations: AGO2—protein argonaute-2; ERα—estrogen receptor α; HER2—human epidermal growth factor receptor 2; miRNAs—microRNAs; MISS—membrane-initiated steroid signaling; mTORC1—mammalian target of rapamycin complex 1; *PGR*—progesterone receptor gene; PgR—progesterone receptor. Created with BioRender.com—accessed date 22 September 2021.

Whereas most estrogenic actions in BC cells seem to be driven by ligand binding to ERα homodimers, the latter may also form heterodimers with ERβ1, which can promote transcription of a distinct pool of genes, and to down-regulate several ERα-dependent genes, including *PGR* (Figure 2) [67,68]. The inverse correlation between ERβcx, a splice variant of ERβ, and PgR was noted; interestingly PgR-low BCs expressing ERβcx showed poorer response to tamoxifen [69].

Expression of PgR is also modulated by splice variants of ERα, e.g., ERα36, which positively correlates with PgR expression [70,71]. In vitro study utilizing ERα36 knock-out cell lines demonstrated reduced levels of PgR and its altered phosphorylation at Ser294 and Ser345 [71].

Additionally, there is a dominant-negative splice variant of ERα (ERαΔ7), which is non-functional, but is detected by IHC. This may explain why a subset of ERα(+) tumors shows the molecular characteristics of the basal subtype [72]. Interestingly, the frequency of PgR expression in ERα(+)/ERαΔ7-high basal carcinomas was 29.7% compared to 85.2% for ERα(+)/ERαΔ7-low luminal B carcinomas [73]. Identification of such hormone receptor variants may in the future support treatment decision-making, but current routine procedures have not incorporated their assessment yet.

### 2.7. MicroRNA (miRNA) Profiles of ERα (+)/PgR(−) Breast Cancers

miRNAs are small non-coding molecules with an average length of 22 nucleotides [74]. They regulate gene expression via the formation of miRNA-induced silencing complex (miRISC), which binds to the 3’UTR of a target gene [75]. Subsequently, translational repression, mRNA destabilization, degradation, and deadenylation occur [75].

The interplay between miRNAs and ERα expression is well described, but still not completely understood. Estrogens bound to ERα regulate miRNA processing and the formation of miRISC interacting with Drosha, DICER, and protein argonaute-2 (AGO2), and in this way influence gene repression by miRNAs [76]. On the contrary, multiple miRNAs modulate the expression and action of ERα via direct interactions with *ESR1* mRNA and alterations of ERα coregulators. Additionally, some oncogenic miRNAs interfere with ERα-dependent signaling pathways, which, in consequence, may result in partial loss of ERα functionality reflected by loss of PgR expression in BC (i.e., acquisition of ER(+)/PgR(−) phenotype).

Recent studies have also shed some light on miRNA regulation of PgR expression. Interestingly, the 3′UTR of *PGR* is the longest amongst mRNAs encoding steroid receptors (9434 nucleotides) but surprisingly contains only six conserved miRNA binding sites. It was demonstrated that exogenous miR-423-5p is capable of inhibiting *PGR* gene transcription in vitro [77], miR-126-3p suppresses PgR expression in mouse mammary gland [78], and miR-181a, miR-23a, and miR-26b down-regulate PgR in ERα(+) BC [79,80]. miR-181a and miR-26 are repressed by estrogen and they belong to the feed-forward loop involving ERα. Their down-regulation following estrogenic stimulation leads to *PGR* up-regulation and their up-regulation in ERα(+) tumors may contribute to ERα(+)/PgR(−) BC development [79]. The main interactions between microRNAs and PgR expression are shown in Figure 2.

Estrogen-dependent PgR up-regulation may be abrogated by progestin-controlled miRNAs, most notably miR-129-2 and miR-513a-5p. Progesterone treatment of BC cell lines leads to the up-regulation of miR-129-2, resulting in down-regulation of PgR, and tumors with elevated miR-129-2 have significantly decreased levels of PgR [81]. Similar effects were observed for miR-513a-5p, which represses PgR expression and reduces the amounts of PgR induced by estrogenic stimulation [82]. In vitro studies demonstrate that inhibitors of miR-129-2 increase expression of PgR providing a potential tool for stabilization of PgR levels in PgR-low/negative patients considered for hormonal therapy [81].

In our recent study, we compared miRNA profiles of two groups of single-steroid-hormone-receptor-positive BC, ER(+)/PgR(−) and ER(−)/PgR(+) [83]. The first group demonstrated elevated levels of miR-30a-5p, miR-29c-3p, miR-141-3p—members of miRNA clusters characterizing ER(+) tumors, and miR-423-5p, whose role in PgR silencing was discussed before [77]. Interestingly, miR-30-5p has previously been shown to suppress PgR expression in BC cell lines [83]. Additionally, the miR-29 family targets and represses transcription of the PgR-regulated gene, *ATP1B1* [82]. Conversely, progestin treatment inhibits the expression of miR-29. miR-141-3p is another miRNA with reciprocal associations with PgR: down-regulation of miR-141-3p increases PgR levels, whereas progestin treatment decreases levels of miR-141-3p [84]. In conclusion, miR-29 and miR-141-3p up-regulation in ER(+)/PgR(−) BC may reflect diminished progestin-dependent signaling in these tumors.

An interesting mechanism of PgR regulation in BC, partially driven by miRNA, involves a model, in which early lesions recapitulate the developmental program of normal mammary gland orchestrated by progesterone signaling via PgR and moderate HER2 expression [85]. This program facilitates the early dissemination of cancer cells, enhancing migration and stemness. Growing lesions gradually increase their tumor cell density and overexpress HER2, which up-regulates the expression of miR-9-5p and miR-30a-5p, leading to the down-regulation of *PGR* in the mouse BC model. This mechanism increases the proliferation of cells contributing to primary tumor growth but impairs its ability to spread. Plausibly, ERα (+)/PgR(−)/HER2(+) BCs show inferior prognosis because they represent an end-point in the pathway beginning with early, occult dissemination initially driven by PgR(+) cells, while clinically overt PgR(−) cancers may comprise only of residual scattered phospho-PgR(+) spots with stem cell potential and an ability to spread [85].

An additional mechanism of PgR regulation by miRNA involves miR-155 and the mTOR pathway. In BC, IGF-mediated mTORC1 activation down-regulates PgR expression [30]. Increased expression of miR-155 in ERα(+) BC cells enhances mTORC1 signaling via inhibition of the mTORC2 signaling component Rictor [86]. TCGA data on BC show that levels of Rictor and PgR positively correlate with each other, whereas Raptor (complexed with mTORC1) shows an inverse correlation with PgR [86]. mTOR inhibitor, everolimus, demonstrated efficacy in combination with ET in advanced BC and is generally believed to reverse endocrine resistance by inhibition of mTORC-1-dependent phosphorylation of ERα, but de-repression of PgR expression may represent another possible mechanism of action [87,88,89]. Nevertheless, limited data suggest that PgR status is not a predictive factor in advanced/metastatic BC treated with everolimus [90].

Curiously, a group of small duplex RNAs, antigene RNAs (agRNAs) are also able to regulate gene expression by targeting gene promoters (noncoding transcripts). Several studies demonstrated that PgR expression is regulated by synthetic agRNAs mediated by argonaute (AGO) proteins, but it was unknown if similar effects may be mediated by endogenous RNAs [91]. A very recent study shows that sequestosome 1 (p62) accumulation in BC cells triggers PgR suppression in an AGO2-mediated mechanism, comprising most likely agRNAs, not miRNAs [92]. On the contrary, in another study, high AGO2 levels were correlated with PgR loss due to altered ERα signaling probably driven by miRNA [93]. If small RNAs can precisely up-regulate expression PgR in BC to increase its sensitivity to ET remains to be elucidated.

## 3. Loss of PgR during Therapy and in Breast Cancer Relapse

A large meta-analysis of steroid HRs discordance in primary and recurrent BCs estimated the frequency of secondary PgR loss at 46% of patients, being more common in distant metastases than in local relapses [23]. The prognostic significance of this conversion is not well established, however, some studies report on the association between worse outcomes and the negative conversion of steroid HRs [12]. The loss of ERα and/or PgR in relapsing tumors or after primary systemic treatment probably indicates the selection of HR-negative cells in a heterogeneous pool of tumor cells. Moreover, circulating tumor cells (CTCs) frequently show discordant profiles with primary tumors. PgR(−) CTCs are present in 68–87% of patients with PgR(+) primary tumor, and this pool may be responsible for ERα(+)/PgR(−) metastases [94]. On the other hand, in metastatic BC, the loss of PgR expression on CTCs may occur, even if still present in both primary tumors and metastases [95].

The switch from PgR(+) to PgR(−) after neoadjuvant chemotherapy occurs in 12–15% of cases and is associated with worse clinical outcomes [96,97]. Similarly, neoadjuvant ET with SERMs or AIs may lead to the down-regulation of ERα and PgR, respectively [12]. A letrozole-induced decrease in PgR expression is most likely due to decreased estrogens levels and diminished estrogenic signaling [98,99]. Accordingly, studies on patient-derived xenografts and cell lines demonstrate that estrogen withdrawal can lead to PgR expression loss [100].

The decline in PgR expression is also promoted in a time-dependent manner by treatment with fulvestrant, as demonstrated in sequential biopsies of advanced BC [94]. Fulvestrant and the other SERDs have no agonistic activity and inhibit ligand binding to ERα, promote its degradation, and diminish transcription of ERα-dependent genes, including *PGR* [101]. Fulvestrant response rate seems independent from the baseline HER2 and PgR status because it antagonizes nuclear, cytoplasmatic, and membrane-bound ERs, completely inhibiting the cross-talk between the growth factor receptor and estrogen signaling [102]. Intriguingly, patients with a retained high PgR expression have a longer duration of response than patients with PgR loss at 6 weeks of treatment [101]. Moreover, overexpression of Tissue Inhibitor of Metalloproteinases-1 (TIMP1) ensues the down-regulation of PgR and drives resistance to fulvestrant in the MCF-7 cell line, but the mechanism of TIMP1-associated PgR depletion is unknown [103]. Resistance to fulvestrant may also be driven by mitogen-activated protein kinase (MAPK) pathway activation with increased levels of ERK, MEK, and RSK, kinases known to phosphorylate and inactivate PgR, hence, potentially, providing space for treatment with antiprogestins [104]. Phase 2 clinical trial investigating the combination of fulvestrant and onapristone for advanced or metastatic BC after progression on aromatase and CDK4/6 inhibitors (NCT04738292) is planned [105].

## 4. Genetic Landscape of ERα(+)/PgR(−) BC

Genomic alterations of ERα(+)/PgR(−) BC have been extensively studied in recent years. In terms of genetic stability, these tumors are characterized by increased DNA copy number gains when compared to double-positive BC cases [16]. Their mutation burden is intermediate between double-positive and triple-negative BCs [31]. In a comprehensive analysis of the large publicly available datasets, ERα(+)/PgR(−) tumors shared 5668 mutated genes with ERα(+)/PgR(+) cancers, while 1319 genes (19%) were uniquely altered in the former group [33]. The most commonly mutated genes were *PIK3CA*, *TP53*, *GATA3*, *CHD1*, *KMT2C*, *MUC16*, *MAP3K1*, *ARID1A*, *AHNAK2*, and *SYNE2* [29]. When compared to double-positive cancers, ERα(+)/PgR(−)/HER2(−) tumors displayed higher *TP53* and lower *PIK3CA* mutation rate, and more frequently showed amplification of oncogenes *ZNF703* and *RPS6KB1* [13,27].

Taking into consideration intrinsic molecular phenotypes, 15–46% of ERα(+)/PgR(−)/HER2(−) BCs are classified as PAM50-defined luminal A tumors, next 29–58% are classified as luminal B, and 20–27% as HER2-enriched or basal [31,106]. When compared to double-positive tumors, ERα(+)/PgR(−) BCs are characterized by lower endocrine sensitivity scores, enriched biosynthesis, metabolism, and DNA replication. The probability of benefits from ET in ERα(+)/PgR(−) tumors may be estimated also from three IHC markers: GATA3, CK5, and EGFR [31].

Analysis of mRNA expression profiles from several datasets demonstrated that ERα(+)/PgR(−) BCs share gene expression patterns both with double positive and double negative tumors [107]. This was confirmed also in our analysis of the TCGA dataset, where we identified 2 and 32 differently expressed genes between ER(+)/PgR(−) and double-positive or double negative tumors, respectively. Importantly, we found only 10 genes uniquely differentiating between two subtypes of single hormone receptor-positive tumors [83].

## 5. The Biology of ERα(+)/PgR(−) BC

**Figure 3:**
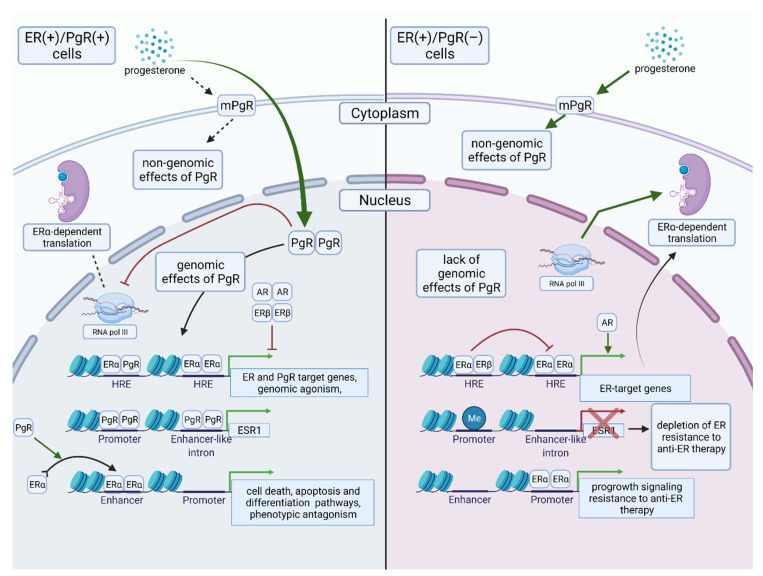
Biology of ERα(+)/PgR(+) and ERα(+)/PgR(−) breast cancer. Green arrows indicate stimulatory effects, red T-shaped lines depict inhibitory effects. In tumor cells co-expressing ERα and nuclear PgR the latter may exert both non-genomic and genomic effects. It regulates the expression of genes in a similar way to ERα (genomic agonism) but guides ERα binding to chromatin to induce expression of genes associated with good outcomes (phenotypic antagonism). PgR interacts with translational machinery (mainly RNA polymerase III) reducing its availability for ERα-dependent translation. Loss of nuclear PgR results in a shift of ERα role from distant enhancer to proximal promoter activating subset of genes associated with cancer progression. Depletion of PgR increases *ESR1* gene promoter methylation and down-regulates *ESR1*. Other steroid receptors, i.e., ER and AR may exert different effects on ERα-dependent genes expression in ERα(+)/PgR(+) and ERα(+)/PgR(−) breast cancers. For details, see text. Abbreviations: AR—androgen receptor; *ESR1*—estrogen receptor 1 gene; ER—estrogen receptor; HRE—hormone receptor element; (m)PgR—(membranous) progesterone receptor, RNA pol III—RNA polymerase III. Created with BioRender.com—accessed date 22 September 2021.

The biology of ERα(+)/PgR(−) BC cells is probably highly variable and depends on many cofactors (Figure 3). Isolated effects of ER (stimulated by estrogens) and PgR (stimulated by progestins) on gene expression are similar because they regulate the expression of shared target genes in the same direction (genomic agonism) [108]. In BC cells positive for both types of steroid hormone receptors, PgR competes with ERα regarding access to RNA polymerase III, and, hence, reduces its availability and ERα-dependent translation [84]. In consequence, when PgR expression is lost, ERα gains access to a broader range of translational machinery, which may promote tumor aggressiveness and growth. Moreover, chromatin binding of ERα is more consistent in double-positive tumors, whereas ERα binding patterns in PgR(−) subset are highly variable [108,109]. In PgR-deficient cells, ERα predominantly binds in the proximity to transcription start sites, whereas in PgR(+) cells PgR redirects ERα to bind distally to promoters. In consequence, in ERα(+)/PgR(−) BC ERα seem to act as a proximal promoter rather than distal enhancer of gene transcription, which stimulates pro-growth estrogenic signaling and reduces the responsiveness to ET [108]. Thus, PgR acts as a molecular rheostat regulating ER activity. Additionally, PgR mediates ERα chromatin binding to genes involved in cell death, apoptosis, and differentiation pathways and blocks ERα-dependent tumor growth [32]. Moreover, unliganded PgR regulates *ESR1* transcription via epigenetic modifications of the *ESR1* promoter. PgR depletion results in *ESR1* promoter hypermethylation, down-regulating expression of ER, which cannot be reversed after PgR re-expression [109].

The combined effect of estrogens and progestins on BC cells co-expressing ERα and PgR demonstrate that there is phenotypic antagonism between ERα and PgR. It has clinical consequences—in premenopausal patients, PgR has a more pronounced positive prognostic significance because of the availability of progesterone, which stimulates PgR signaling [110]. On the contrary, in post-menopausal females, progesterone levels are low, and thus are unable to produce a prominent phenotypic antagonism to ERα, which makes PgR expression a less important predictive factor in older patients.

Once PgR expression is lost, other receptors such as ERβ or androgen receptor (AR) may more significantly modulate ER-dependent actions. In the absence of PgR, AR most likely enhances ER-mediated transcription. In the nuclei of ER(+)/PgR(+) BC cells, AR competes with ER and PgR to bind to DNA, thus interfering with the estrogen-mediated transcription. Conversely, when PgR is lost, another receptor, ERβ, down-regulates ERα target genes, whereas AR enhances ERα target gene transcription and potentially contributes to tumor growth [111]. However, high AR expression is associated with prolonged relapse-free survival, lower grade, and lower number of affected lymph nodes in ERα(+)/PgR(−) BC, thus the mechanistic role of AR and its influence on ERα(+)/PgR(−) tumor aggressiveness requires further studies [112,113].

The loss of nuclear PgR expression does not imply loss of progestin responsiveness in BC cells [114]. Similarly to estrogens, progestins may act via membrane receptors (mPgRs), which have three subtypes: mPgRα, mPgRβ, and mPgRγ, the first being the most prevalent in breast tissue [115]. In PgR(−) BC cell lines progesterone produces an antiapoptotic response and activates MAPK and PI3K/Akt through mPgRs [114,116]. Expression of mPgR was correlated with HER2-overexpression, a number of lymph node metastases, and a worse prognosis in BC [117]. Thus, mPgRs might be important players in the biology of ERα(+)/PgR(−) BCs providing pro-growth signals. Nevertheless, some in vitro studies utilizing BC cell lines demonstrated that mPgRα mediates antiproliferative and antimetastatic signaling of progesterone [118,119], although the effects of mPgRs are potentially dependent on the model (in vitro vs. in vivo or clinical studies), progesterone levels, and competition with nuclear receptors. Of note, there is an inverse relationship between nuclear PgR and mPgR [117].

A recent study in PgR-low/null tumors defined phospho-PgR target gene sets (*ERBB2*, *PAX2*, *AHR*, *AR*, and *RUNX2*) which regulate cancer stem cell biology and increase tumor heterogeneity [85]. Paradoxically, antiprogestin treatment may possibly be effective in these clinically PgR(−) tumors, preventing the development of endocrine resistance [85]. However, not all antiprogestins are equally adequate to this approach, since it was shown that in the presence of progesterone onapristone blocks Ser294 phosphorylation, whereas mifepristone and aglepristone induce Ser294 phosphorylation, behaving similar to partial agonists of PgR [85]. Phase I study of onapristone in heavily pre-treated, metastatic endometrial, ovarian, and BC showed promising results and proposed activated progesterone receptor as a potential predictive factor [120].

The understanding of PgR significance in BC is further complicated by the coexistence of its isoforms, as phosphorylated PgR-A is a more potent driver of cancer stem cell expansion, whereas PgR-B is involved in BC cells proliferation [121]. In normal mammary gland tissue, the levels of PgR-A and PgR-B are similar, while the ratio is disturbed during cancer transformation, usually resulting in PgR-A prevalence [122]. In vitro studies demonstrated that the PgR-A/PgR-B ratio determines the functional outcome of PgR action, including both the target genes and response to hormones and growth factors [123]. This observation was further confirmed in clinics because a high PgR-A/PgR-B ratio was indicative of a shorter time to relapse in patients treated with tamoxifen within the ATAC trial [124]. Interestingly, it is speculated that tamoxifen resistance and the worse prognosis are associated solely with methylation of *PGRA* promoter, resulting in the functional predominance of PgR-B [57]. High frequency of ERα:PgR-B interaction was predictive of relapse on an adjuvant AI, and in some cases, a substantial amount of ERα:PgR-B interactions coexisted with a lack of IHC-detectable PgR expression [125].

It was recently shown that among HER2-negative tumors ERα(+)/PgR(−) BCs display distinctive tyrosine kinases profiles [126], characterized by higher overall kinase activity than double-positive tumors, with RAS, PI3K, and ErbB signaling being mostly responsible for these differences. Four kinases showed significant expression differences between PgR(−) and PgR(+) tumors: fibroblast growth factor receptor 4 (FGFR4) and LCK were up-regulated, whereas Fyn-related kinase (FRK) and macrophage-stimulating 1 receptor (MST1R) down-regulated in PgR(−) cases. Interestingly, all these kinases are directly regulated by progesterone. Moreover, Tahiri et al. identified 24 kinase-encoding genes differentially expressed between double-positive and PgR(−) tumors, dividing ER(+)/HER2(−) BCs into two prognostically distinct clusters: cluster 1 comprising mostly PgR(+) patients with a better prognosis, and cluster 2 characterized by worse prognosis and the predominance of PgR(−) patients [126]. Additionally, PgR(−) patients in cluster 2 had inferior survival to PgR(−) patients in cluster 1. Unfortunately, the association between the clusters and luminal A vs. B phenotype was not studied. Importantly, these associations are not seen in HER2(+) samples, suggesting that the effects of HER2 are dominant. This is further supported by our study on single hormone receptor BC, in which miRNA profiles of single hormone receptor-positive breast cancers were mainly dependent on the status of HER2, rather than on ERα/PgR status [83].

## 6. Conclusions

Lack of PgR expression in ERα(+) BC has multiple potential explanations but the molecular, pathological and clinical heterogeneity of this group remains underappreciated. The biology of ERα(+)/PgR(−) BC is context-dependent, being highly modulated by the cross-talk between growth factors receptors and nuclear or membranous steroid hormone receptors. Novel therapeutic targets as microRNAs, epigenetic modifications, tyrosine kinases, and transcriptionally overactive PgR should be further investigated in the future. Identification of the mechanism of PgR loss in each patient seems challenging, yet it may provide important information on the biology of the tumor and predict its responsiveness to the therapy. Finally, future studies should focus on the investigation of novel biomarkers predicting the disease course, as well as its response to endocrine and chemotherapy in this distinctive group of patients.
